# Transcriptional analysis identifies key genes involved in metabolism, fibrosis/tissue repair and the immune response against *Fasciola hepatica* in sheep liver

**DOI:** 10.1186/s13071-015-0715-7

**Published:** 2015-02-25

**Authors:** Cristian A Alvarez Rojas, Brendan RE Ansell, Ross S Hall, Robin B Gasser, Neil D Young, Aaron R Jex, Jean-Pierre Y Scheerlinck

**Affiliations:** Centre for Animal Biotechnology, Faculty of Veterinary and Agricultural Sciences, The University of Melbourne, Parkville, Victoria 3010 Australia; Faculty of Veterinary and Agricultural Sciences, The University of Melbourne, Parkville, Victoria 3010 Australia

**Keywords:** *Fasciola hepatica*, Fascioliasis, Sheep, Liver, Transcriptomic, Molecular responses in the host

## Abstract

**Background:**

Although fascioliasis has been relatively well studied, little is known about the molecular basis of this disease. This is particularly relevant, considering the very different response that sheep have to *Fasciola hepatica* relative to cattle. The acute phase of this disease is severe in sheep, whereas chronic fascioliasis is more common in cattle.

**Methods:**

To begin to explore the host-response to *Fasciola* in sheep and improve the understanding of the host-pathogen interactions during the parasite’s migration through liver parenchyma to the bile duct, we used RNA sequencing (RNA-seq) to investigate livers from sheep infected for eight weeks compared with those from uninfected controls.

**Results:**

This study identified 572 and 42 genes that were up- and down-regulated, respectively, in infected livers relative to uninfected controls. Our molecular findings provide significant new insights into the mechanisms linked to metabolism, fibrosis and tissue-repair in sheep, and highlight the relative importance of specific components of immune response pathways, which appear to be driven toward a suppression of inflammation.

**Conclusions:**

This study is, to our knowledge, the first detailed investigation of the transcriptomic responses in the liver tissue of any host to *F. hepatica* infection. It defines the involvement of specific genes associated with the host’s metabolism, immune response and tissue repair/regeneration, and highlights an apparent overlapping function of many genes involved in these processes.

**Electronic supplementary material:**

The online version of this article (doi:10.1186/s13071-015-0715-7) contains supplementary material, which is available to authorized users.

## Background

*Fasciola hepatica* is a parasitic flatworm (class Trematoda) responsible for liver fluke disease or fascioliasis in various mammals. This parasite occurs mainly in temperate climatic regions, where suitable conditions favour the survival of particular aquatic snails (Lymnaeidae) that act as the intermediate host [1]. Fascioliasis affects livestock, including sheep and cattle, causing major financial losses due to morbidity and mortality [2-5]. Humans can also become infected by *F. hepatica* or *F. gigantica*, with > 90 million people being at risk of infection worldwide [6]. Despite its impact, this disease remains neglected, in terms of control and research efforts [7,8].

*Fasciola hepatica* is transmitted to the mammalian host via the ingestion of the infective stages (metacercariae) which are usually encysted on aquatic vegetation. Following passage through the stomach(s) and upon entry to the small intestine, the metacercariae excyst and the newly excysted juveniles (NEJs) penetrate the small intestinal wall, to then migrate through the abdominal cavity in search of the liver. The juveniles penetrate the liver capsule and migrate through the parenchyma to the major bile ducts, a process mediated by the secretion of a complex mix of digestive enzymes produced by the parasite, including cathepsins and other cysteine/serine proteases [9-12]. After 6–8 weeks, the immature flukes reach the bile ducts, where they mature to adults and live for years [1].

The severity of fascioliasis is largely influenced by the infectious dose, and the age and immune status/response of the host [3]. The disease is divided into acute (1–6 weeks after infection) and chronic (from 7–8 weeks) phases, each of which has been explored extensively using histopathological methods [13-15]. During the first two weeks of infection in sheep, the liver is usually congested, with fibrous tags on its surface and haemorrhagic tracks in the parenchyma, mainly in the left lobe [3]. Around the fourth to fifth week after infection, the migratory tracks become yellow and are surrounded by haemorrhage. Tissue atrophy may be observed in some lobes, and fibrin tags as well as fibrous/sclerotic scarring (i.e. wound repair/healing) are usually apparent [3]. Between the sixth and eighth weeks of infection, flukes are 6 mm to 10 mm in size and tend to localize in the left lobe, while some of them start to enter the bile ducts [3]. At this stage, infected animals might present with anaemia [16], hypoalbuminaemia [17], eosinophilia [18] and/or hypoglycaemia [17]. Fibrosis appears from the fourth week after infection, and gradually increases as healing occurs in older migratory tracks. Upon maturation in the bile duct, adult flukes may persist for several years, causing fibrosis/sclerosis of the duct (being often more pronounced in cattle than sheep) and inappetence, productivity losses and/or failure to thrive [3,13].

Despite extensive research of fascioliasis, little is known about the interactions between *F. hepatica* and its mammalian hosts on a molecular level. Nonetheless, studies of the secreted proteome [19-22] and transcriptome [23] of adult *F. hepatica* have provided some insights into the pathogenesis of fascioliasis. In addition, investigations of major parasite-derived secreted proteins have shown that cathepsins and other secreted proteases [24,12,25,26,10], immunomodulatory/anti-inflammatory proteins, such as thioredoxin peroxidase [27-30] and helminth defence molecules (FhHDM-1) [20,31,32], are intimately involved in the parasite-host interplay. On the other hand, little is known about the impact of *Fasciola* at the cellular biological and physiological levels. Cellular changes in the infected liver are associated with a disruption of the mitochondrial electron transport chain, and a substantial loss of cytochrome P450 activity and glycogen content in the left lobe [3]. Physiological changes in infected animals can include glycaemia [33], lipidaemia [34], a reduction in plasma ascorbic acid [35] and testosterone catabolism [36] as well as an increase in iron and iron-binding capacity [35], and a variable effect on coagulation [37]. However, the specific molecular mechanisms underpinning these changes are not well understood.

From an immunological perspective, although it has been reported that the host response to *Fasciola* infection is primarily Th2-driven [38,39], the precise components of the immune system that are activated or potentially suppressed in the host during *F. hepatica* infection are not known. Similarly, although there is histological evidence of scarring and wound healing in the liver following acute fascioliasis in sheep [3], the key genes involved in this process have not been identified. The availability of the sheep genome [40] now provides an excellent opportunity to explore host responses to *Fasciola* infection at the molecular level. In the present study, we characterized the sheep response to *Fasciola* infection by exploring transcriptional changes in the livers of sheep during the early phase of *F. hepatica* infection.

## Methods

### Animals and experimental design

All experiments were approved by the Ethics Committee of the Faculty of Veterinary and Agricultural Sciences of the University of Melbourne. Eight helminth-free lambs (<6 months of age) were housed in raised pens at the animal facility of the University of Melbourne. At two weeks, four animals (the ‘infected’ group) received a single oral dose of 180 metacercariae of *F. hepatica* purchased from Baldwin Aquatics Inc., USA. Four additional sheep of the same age and genetic background were maintained as controls and kept in a separate, raised pen in the same facility. All feed, water and management practices were consistent for all infected and control sheep throughout the entire study period. Individual sheep were clinically monitored on a weekly basis. A blood sample was obtained from the jugular vein of each animal twice per week to undertake white blood cell (WBC) differential count in blood smears. Eosinophil levels were used to assess the progression of the infection. Fresh faeces (10 g) were collected on alternate days, six weeks after infection, using a sterile collection bag (attached to the rear of the animal), and tested for the presence of helminth eggs using a standard approach [41]. Eight weeks after infection, each animal was euthanized by intravenous injection of pentobarbitone sodium (172.5 mg/kg) and immediately necropsied. Upon necropsy, multiple 1 cm^3^ samples containing lesions characteristic of fascioliasis (tracks) were collected from each animal, snap frozen in liquid nitrogen and stored at −80°C until further use for RNA extraction. Some of these samples were fixed in formalin and, stained with haematoxylin and eosin (H&E) for histopathological examination. Each stained tissue sample was examined by microscopy at 400× magnification, and eosinophils were enumerated in 10 randomly-selected fields of view.

### RNA extraction and library preparation

Total RNA was extracted from 50 mg of fresh-frozen liver tissue by TriPure reagent (Roche Diagnostics), following the manufacturer’s instructions, and resuspended in RNAse-free water (Life technologies). Purified RNA was treated with Turbo DNase (Life Technologies). RNA quality was evaluated using a Bioanalyzer 2100 (Agilent, USA); only samples with an RNA quality indicator (RQI) of ≥8 were used for sequencing. Following quality assessment, polyadenylated (polyA+) RNA was purified from 10 μg of total RNA from each sample using Sera-Mag oligo (dT) beads (GE Healthcare, USA), fragmented to 300–500 bp, reverse-transcribed using random hexamers, end-repaired and adapter-ligated, according to the manufacturer’s protocol (Illumina). Ligated products of ~400 bp were excised from agarose and PCR-amplified (15 cycles). Products were purified over a MinElute column (Qiagen) and subjected to paired-end RNA-seq (read length: 125 nt) using HiSeq 2000 Ilumina (Yourgene Bioscience, Taiwan).

### RNA-seq analysis

Paired-end RNA-seq reads for each sample/replicate were filtered for quality and adapter-trimmed using Trimmomatic software [42] (sliding window: 4 bp, leading and trailing: 3 bp, minimum read length: 100 bp; Phred quality: 25). Filtered reads were subjected to K-mer correction using the program corrector [43]. Data have been deposited in the Sequence Read Archive (SRA) from the National Center for Biotechnology Information (NCBI; Bio Project ID: PRJNA266851). All filtered reads were mapped to the latest assembly of the sheep genome (sheep genome: Oar v3.1, available under GenBank accession code GCA_000298735.1 [40]), and the transcription abundance of each annotated gene (according to the Ensembl [44] annotation for this version available at http://e74.ensembl.org) quantified using the program RSEM [45]. The transcription abundance of each gene in each sample was represented as transcripts per million reads (TPM) [46]. Differentially transcribed genes (DTG) were identified in R [47] using the NOISeqbio package (Tarazona et al., 2011), incorporating a between-samples trimmed-mean of M-value (TMM) normalization using a probability of differential expression (q) = 0.9. In NOISeqbio, q is equivalent to 1-FDR (false discovery rate), where FDR is considered as an adjusted p-value. Genes with TPM value = 0 were replaced with a value = 0.05 (k) for NOISeqbio analysis [48]. We then focused on genes differentially transcribed by more or less than 2-fold (i.e., ≥ 2-fold change in TPM values for at least three replicates per group).

### Bioinformatic analyses

Enriched gene ontology (GO) terms for up- and down-regulated genes were assigned using the BinGO plug-in for Cytoscape [49]. Briefly, the Ensemble identification code (ID) of each sheep transcript predicted with reference to the genome was used to obtain the orthologous gene name for each specific transcript in the *Homo sapiens* genome database (GRCh37.p13) using Biomart [50], as described previously [51]. The gene names obtained were used as input in BinGO with the most updated version (released 10th July 2014) of the human genome annotation as a reference (GO consortium). For DTG clusters, significantly over-represented (i.e. enriched) GO terms within the ‘biological process’, ‘molecular function’ and ‘cellular component’ categories were identified using hyper-geometric mean tests (p < 0.05 after FDR correction). Similarly, to discover enriched pathways and protein family hierarchies for up- and down-regulated DTGs, close homologues of sheep transcripts were identified in the KEGG database using BLAST [52], using an expected value of 10^−5^, from which K (pathway) and Br (Brite) terms for each gene were inferred. Fisher hyper-geometric mean tests were then performed to identify significantly enriched KEGG pathways and enzyme hierarchies for DTGs relative to all KEGG-annotated genes [53,54]. In addition to a broad functional characterization of KEGG pathways and GOs, we also investigated the transcriptional abundance of genes with functions previously implicated in liver fibrosis or the host immune responses against helminths.

## Results

### Experimental infection

**Figure 1:**
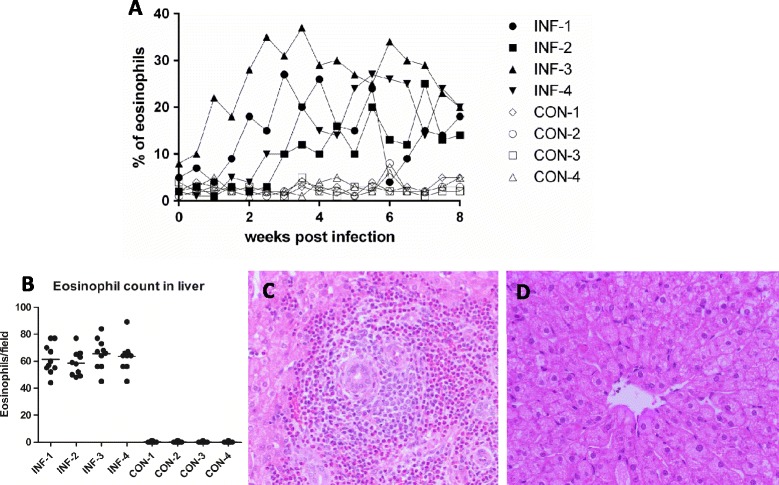
**Eosinophil count in blood and histological sections of liver tissue from infected and control sheep. (A)** Eosinophil count (% of total white blood cells) after microscopic examination of peripheral blood smears from the infected (INF-1, INF-2, INF-3 and INF-4) and control (CON-1, CON-2, CON-3 and CON-4) sheep. Normal range for eosinophils in sheep: 1-10%. **(B)** Average of eosinophil count from 10 fields at 400X in histology sections of each biological replicate from the infected and control groups. **(C)** Typical microscopic appearance (400X) of liver from the infected sheep at 8 weeks post infection, massive infiltration of inflammatory cells and deposition of collagen can be observed. **(D)** Normal architecture of the liver from control animals.

Over the eight-week experimental infection period, we detected a statistically significant increase in the relative number of eosinophils vs. the total of white blood cells in all infected sheep when compared with uninfected controls (Student's *t*-test; p-value: 0.05). All sheep in the infected group had an eosinophil count exceeding 10% of the total WBC count (range: 12-39%) from the fourth week after infection compared with controls which had counts of 3% (range: 1-5%) at the same time point. At no point during the experiment did eosinophil counts in the control sheep exceed the maximum reference level for uninfected sheep (10% of the total WBC [55]). In contrast, from four weeks after infection, at no point did eosinophil counts drop below 10% of the total WBC count in the infected sheep, with the exception of animal INF-1, which presented an eosinophil count of 4% at week six (Figure 1A). In the liver, eosinophil numbers from infected animals ranged between 42 and 89 cells per field of view (400×) (Figures 1B and C), whereas no lesions or notable infiltration of eosinophils or other types of WBC were observed in liver tissue samples collected from uninfected control sheep (Figure 1B and D).

From the sixth week after infection until the end of the experiment, daily faecal examination was conducted on each infected and control animal. No parasite eggs of any species were detected in any animal. The sheep showed no overt clinical signs of infection (e.g., ill thrift, anaemia or weight loss) in either infected or control groups during the experiment. However, upon necropsy, direct examination of the livers showed clear signs of fascioliasis in all infected sheep, including parenchymal tracks characteristic of migrating juvenile parasites and evidence of haemorrhage in several lobes of the liver. Neither comparable pathological signs nor noticeable evidence of damage to the liver tissue was observed in any of the control sheep.

### RNA-seq pre-processing, mapping and differential transcription analysis

After trimming and correction, a total of 82,557,944 RNA-seq paired-end reads were generated from all cDNA libraries (mean of 13.2 million per sample). Alignment rates to the sheep genome ranged from 63.2 to 71.2% per sample, with an average coverage of 32.2X (range 13X-65.6X) (see Additional file 1). At a lower TPM threshold of 0.05, the mean transcript detection was 14,091 for the *Fasciola*-infected sheep (range: 13,413-15,087) and 12,375 for uninfected controls (range: 11,157-13,937). These data allowed differential transcription to be assessed among 12,934 genes in the liver tissue samples, of which 1,012 were differentially transcribed genes in total (820 up-regulated and 192 down-regulated in infected relative to control animals). Further refinement of DTGs to select for the most robust transcriptional differences (≥ two-fold difference in TPM values for at least three of four infected sheep relative to the uninfected control mean) retained 572 up-regulated and 42 down-regulated genes representing 4.8% and 0.24% of all detected transcripts respectively (see Additional file 2).

### Gene ontology and KEGG analysis

Of the up- and down-regulated genes identified in *Fasciola*-infected sheep, 492 and 31 were assigned one or more predicted GO term(s) respectively. Of these GO terms, 211 and 121 were significantly enriched (hyper-geometric test; FDR = 0.05) within up- and down-regulated genes respectively (see Additional file 3). For the down-regulated genes, enriched GO terms related to the molecular function ‘oxidoreductase activity’ involving cholesterol, flavin, alkane, leukotriene, linoleic and arachidonic acid were overrepresented (Table 1). ‘Arachidonic acid binding process’ was also overrepresented in up-regulated genes (see Additional file 3: Table S5). For the up-regulated genes, enriched biological process GO terms related to the immune system, including neutrophil aggregation and chemotaxis, regulation of Th2 cell differentiation, regulation of B cell activation; whereas production of α, β and type III interferon was overrepresented among down-regulated genes (Table 1). Up-regulated genes also showed enrichment for GO terms in the ‘cellular component’ category, specifically related to extra cellular matrix (ECM), proteinaceous ECM and collagen type I (Table 1). Importantly, an overrepresentation of GO terms within the biological process category related to the cell cycle and mitosis was observed among up-regulated genes in infected liver (see Additional file 3: Table S4).

**Table 1:** **Overrepresented Gene ontology (GO) terms for up- (↑) and down- regulated (↓) genes in infected liver**

**Major Function**	**Transcription**	p-value *
**Oxidoreductase activity**		
cholesterol 7-alpha-monooxygenase activity (GO:0008123)	↓	0.0017
flavin-containing monooxygenase activity (GO:0004499)	↓	0.0086
leukotriene-B4 20-monooxygenase activity (GO:0050051)	↓	0.0069
arachidonic acid 11 12-epoxygenase activity (GO:0008405)	↓	0.0017
arachidonic acid 14 15-epoxygenase activity (GO:0008404)	↓	0.0017
alkane 1-monooxygenase activity (GO:0018685)	↓	0.0086
linoleic acid epoxygenase activity (GO:0071614)	↓	0.0017
**Immune system**		
neutrophil aggregation (GO:0070488)	↑	0.0007
neutrophil chemotaxis (GO:0030593)	↑	0.0004
regulation of T-helper 2 cell differentiation (GO:0045628)	↑	0.0015
regulation of B cell activation (GO:0050864)	↑	0.0007
negative regulation of B cell apoptosis (GO:0002903)	↑	0.0015
cellular response to type I interferon (GO:0071357)	↓	0.0017
positive regulation of interferon-alpha production (GO:0032727)	↓	0.0001
positive regulation of interferon-beta production (GO:0032728)	↓	0.0006
regulation of type III interferon production (GO:0034344)	↓	0.00001
**Extracellular matrix**		
collagen type I (GO:0005584)	↑	0.0007
proteinaceous extracellular matrix (GO:0005578)	↑	0.0002
extracellular matrix (GO:0031012)	↑	0.0007

*p-value < 0.05.

Analysis revealed GO terms related with oxidoreductase (‘molecular function’), immune system (‘biological processes’) and extra cellular matrix (‘cellular component’).

Full list of GO analyses can be seen in Additional file 3.

Based on conserved orthologues defined by KEGG, 191 of the up-regulated and 19 of the down-regulated genes identified in the infected animals were assigned to one or more conserved biological pathways. For down-regulated genes, we identified an enrichment for KEGG pathways involved in signal transduction (Jak-STAT, TNF, NF-kappa β and PI3-Akt signalling pathways), and NF-kappa β was also enriched for up-regulated genes (Table 2). For signalling molecules and interactions, the cytokine-cytokine receptor interaction was significantly enriched for up- and down-regulated genes, while ECM-receptor interaction was enriched for up-regulated genes (Table 2). An analysis of up-regulated genes also identified an enrichment of KEGG pathways involved in glycan biosynthesis of chondroitin/dermatan sulphate as well as in the metabolism of lipids (ether lipid, arachidonic acid, linoleic acid and glycerophospholipid) and amino acids (valine, leucine and isoleucine biosynthesis, as well as histidine and glutathione metabolism) (see Additional file 4). For down-regulated genes in infected livers, enriched KEGG pathways were associated with a number of metabolic functions, such as primary bile acid and steroid hormone biosynthesis, arachidonic and linoleic acid metabolism, fatty acid degradation, drug metabolism-cytochrome P450, methane metabolism and oxidative phosphorylation. Notably, a number of KEGG pathways linked to the host immune-system were enriched for down-regulated genes, including RIG-I-like receptor signalling, Cytosolic DNA-sensing and Toll-like receptor signalling (see Additional file 4). However, components of the Toll-like receptor signalling pathway were also found to be enriched in up-regulated genes (see Additional file 4).

**Table 2:** **Enriched KEGG pathways for up- (↑) and down-regulated (↓) genes in infected liver parenchyma**

**Pathway/ID**	**Genes**	**Transcription**	p-value *
**Signal transduction**			
Jak-STAT signalling pathway (ko04630)	STAT1, GHR	↓	0.004
TNF signalling pathway (ko04668)	RPS6KA5	↓	0.021
NF-kappa B signalling pathway (ko04064)	DDX58	↓	0.025
NF-kappa B signalling pathway (ko04064)	LAT, CCL4, RELB, TNF, TNFSF14, TLR4, IGLL1, TICAM1	↑	0.009
PI3K-Akt signalling pathway (ko04151)	GHR, C-MET	↓	0.039
**Signalling molecules and interaction**			
Cytokine-cytokine receptor (ko04060)	TNFSF10, GHR, C-MET	↓	0.001
Cytokine-cytokine receptor (ko04060)	CXCR2, ACKR3, INHBA, CCL4, IL1R2, TNF, TNFSF12, CCL3, TGFB1, PF4, TNFSF14, CSF3R	↑	0.027
ECM-receptor interaction (ko04512)	OST, COL1A2, THBS2, VWF, COL6A3, HMMR, COL1A1	↑	0.007

*p-value < 0.05.

Analysis revealed KEGG pathways related with signal transduction and signalling molecules and interaction.

Full list of KEGG pathways analyses can be seen in Additional file 4.

KEGG Brite protein families were also enriched, with cytochrome P450, cellular antigens and cytokines as well as enzyme-linked-receptors representing down-regulated genes. For up-regulated genes, enriched Brite protein families included genes relating to ‘chromosome’ and ‘exosome’, as well as DNA repair/replication proteins, ‘cellular antigens’, ‘cytoskeleton proteins’, ‘cytokines’, ‘enzymes’, ‘lectins’, ‘cell adhesion molecules and their ligands’, and ‘heparan sulphate/heparin binding proteins’ (Table 3).

**Table 3:** **Summary of KEGG Brite ontology analysis for up- (↑) and down- regulated (↓) genes in infected liver**

**Brite definition**	**Brite ID**	**Transcription**	p-value*
Cytochrome P450	ko00199	↓	0.00001
Cytokine receptors	ko04050	↓	0.0041
Enzyme-linked receptors	ko01020	↓	0.0082
Cellular antigens	ko04090	↓	0.0492
Chromosome	ko03036	↑	0.00000001
DNA repair and recombination proteins	ko03400	↑	0.00003
DNA replication proteins	ko03032	↑	0.00018
Cellular antigens	ko04090	↑	0.00036
Cytoskeleton proteins	ko04812	↑	0.00064
Exosome	ko04147	↑	0.0074
Cytokines	ko04052	↑	0.0088
Enzymes	ko01000	↑	0.023
Lectins	ko04091	↑	0.034
Cell adhesion molecules and their ligands	ko04516	↑	0.037
Heparan sulphate/heparin binding proteins	ko00536	↑	0.045

*p-value < 0.05.

### Differential transcription for immune response and liver fibrosis

**Figure 2:**
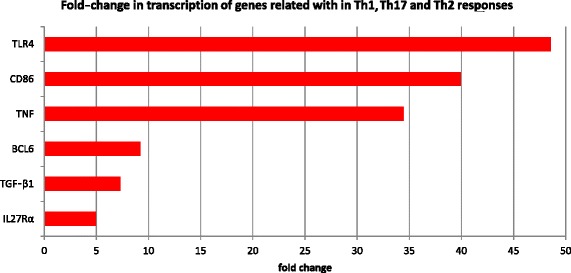
**Change in transcription of genes involved in Th1/Th17 and Th2 immune responses.** Legend: Names on vertical axis represent proteins encoded by these genes. Fold-change on the horizontal axis was calculated using averaged TPM values for infected and control groups of sheep. TLR4: Toll-like receptor 4, CD86: CD86 antigen, TNFα: tumour necrosis factor alpha, BCL6: B-cell CLL/lymphoma 6, TGF-β1: transforming growth factor-beta 1 and IL27Rα: interleukin 27 receptor, alpha.

**Figure 3:**
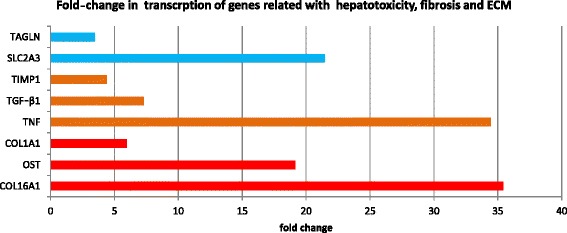
**Change in transcription of genes involved in hepatotoxicity (blue bars), fibrosis (orange) and extracellular matrix formation (ECM) (red).** Legend: Names on vertical axis represent proteins encoded by these genes. Coloured bars indicate the fold change between TPM values for infected compared with control sheep. Fold-change on the horizontal axis was calculated using averaged TPM values for infected and control groups. TGLN: transgelin, SLC2A3: solute carrier family 2 member 3, TIMP1: tissue inhibitor of metalloproteinases 1, TGF-β1: transforming growth factor beta 1, TNF: tumour necrosis factor, COL1A1: collagen, type I, alpha 1, OST: osteopontin and COL16A1: collagen, type I, alpha 1: collagen, type XVI, alpha 1.

Considering the observed link between DTGs associated with infected livers as well as GO terms and KEGG pathways relating to the function and regulation of the host immune system, we specifically queried our differential transcriptional results for genes known to be involved in the Th1, Th2 and Th17 immune responses in mammals (Figure 2). Among the notable genes up-regulated in infected sheep were those encoding BCL6 (B-cell CLL/lymphoma 6) and CD86 (immunoglobulin superfamily), IL1R2 (Interleukin 1 Receptor, Type 2), IL18BP (Interleukin 18 Binding Protein), IL27RA (Interleukin 27 Receptor, Alpha), TGFß1 (transforming growth factor-beta 1), TNF (tumour necrosis factor) and TLR4 (Toll-like receptor 4). Although these genes are commonly recognised as markers of a T-cell response, it is not possible to determine the specific nature of the T-cell response with the data presented here. Interestingly, only three interleukins-related genes (receptor and antagonists) were found to be transcribed in this study, namely IL27RA, IL18BP and IL1R2. Finally, we curated DTGs involved in liver tissue damage (i.e. hepatotoxicity) and repair (i.e. ECM formation and fibrosis). In total, eight genes relating to hepatotoxicity, fibrosis and ECM formation were shown to be up-regulated (Figure 3).

## Discussion

In this study, we provide the first comprehensive transcriptomic investigation of sheep livers during *F. hepatica* migration compared with those of uninfected controls, and identify a number of differentially transcribed genes. Each sheep in the ‘infected’ group had eosinophilia, consistent with that of *F. hepatica* infection for similar time periods in previous reports [56,57,18]. Moreover, eight weeks after infection, the livers of infected sheep showed macroscopic lesions which were consistent with those caused by *F. hepatica* [3]. No comparable evidence of parasitic infection was seen in any of the control sheep. Upon RNA-seq analyses, we detected transcription for 15,966 genes, of which 12,934 were sufficiently transcribed to allow the inference of differential transcription between infected and uninfected controls. Current estimates of the numbers of genes transcribed in mammalian tissues, including liver, range from 10,000 to 15,000 [58,59]. Coulouarn *et al*. [60] identified 12,638 non-redundant transcript clusters in human liver. Our data indicate that we sequenced to a sufficient depth to capture most transcriptional changes in the present study.

Importantly, this study explores transcription in a small, but representative portion of liver tissue corresponding to macroscopic lesions (linked to fibrosis/eosinophilia upon histological observation) at a very specific time-point (8 weeks after infection). An interpretation of these differential transcription data needs to consider the difficulties posed by the complexities of the host-parasite relationship and the liver parenchyma itself. The liver plays a central role in the physiology of the host, being responsible for most amino acid, carbohydrate and lipid metabolism, urea synthesis, detoxification, ketogenesis, albumin and glutathione synthesis [61], and it is also an important organ with respect to innate immunity [47]. Liver contains numerous cell types, including sinusoidal endothelial, hepatic stellate (HSC), liver parenchymal (hepatocytes), Kupffer, natural killer and natural killer T cells [62]. It must also be acknowledged that the tissue sections that we have assessed include cells directly interacting with the parasite and those that might have been affected by the pathogen in an indirect manner. The responses of these individual cell populations have thus been combined in our study and represented as ‘liver tissue’. As the physiology of the liver is complex, subsequent studies might consider undertaking RNA-seq of single cells [63] from populations of individual cell-types sorted by flow cytometer. In addition, although we have presented the data for immune- and fibrosis-related genes in a separate form, it is currently accepted that the immune system acts, in part, through regulating other processes, such as tissue repair and fibrosis [64]. Therefore, some overlap between these two functions might exist for some genes, and the present data cannot ascribe predominance of one function over another. Nonetheless, in assessing the inferred function of these genes, we see a number of key themes, with a significant focus on genes associated with three major functional categories: (a) metabolism, (b) the immune system and (c) response to injury (e.g., liver fibrosis).

Considering the vital role of liver in homeostasis and the overall metabolism of animals, losses in productivity in infected livestock as a consequence of *Fasciola* infection likely relate, at least in large part, to metabolic perturbation [65]. Behm [3] described a significant reduction in the activity of the mitochondrial electron transport chain in infected compared with uninfected, animals including a loss of cytochrome P450 activity. A number of studies support this finding, showing a reduced capacity to metabolize drugs (a major function of cytochrome P450 [66]) in livers infected with *F. hepatica* [67-70]. Consistent with this, in the present study, three genes (*cyp7A1, cyp4a11* and *cyp2j2*) linked to cytochrome P450 were found to be down-regulated in infected sheep.

We note additional, key changes in the metabolism of infected sheep livers in the present study. KEGG analysis revealed enrichment for genes up-regulated in response to *F. hepatica* infection of pathways related to lipid, nucleotide, amino acid, carbohydrate and glycan metabolism (see Additional file 4). This finding is consistent with previous haematological studies of *Fasciola* infection, which have reported changes in glycaemia [33] and lipidaemia [34] in affected animals relative to uninfected controls. A cardinal sign of fascioliasis is anaemia, particularly as a consequence of a large fluke burden, which often significantly affects the health and productivity of animals. Several studies have shown evidence supporting the significant negative effect that *Fasciola* has on metabolic mechanisms associated iron and iron-binding capacity [35], coagulation [37] and/or various biochemical parameters in blood [71], providing insights into the consequences of and responses to major blood loss in infected animals. In the present study, we infected sheep with a relatively small number (n = 180) of metacercariae. Nonetheless, we found a significant increase in the transcriptional abundance of haemoglobin-related genes in infected animals, including haemoglobin subunits α (*hbα*), β (*hbβ*) and foetal subunit β (*hbβf*) (see Additional file 2). These findings suggest an increased abundance of reticulocytes circulating in infected livers, likely due to blood loss caused by the migrating flukes. Interestingly, the up-regulation of four genes predicted to be involved in Fanconi anaemia (*fancc*, *fancd2, fance* and *fancf*), a rare genetic disorder associated with haematological abnormalities in humans [72], were found in infected livers. To our knowledge, this is the first molecular report of the early response of the host to the blood loss with a low intensity infection.

Immune cells that migrate into the infected liver parenchyma include macrophages, eosinophils, neutrophils and lymphocytes [73,56,14,74,75]. The cellular infiltration relates to complex pathways that enable the migration of immune cells from blood to tissues. GO analysis showed that amongst the genes differentially up- or down-regulated, many were associated with immune mechanisms (Table 2). More specifically, genes involved in the innate immune response and, in particular type I interferon production, were down-regulated. In contrast, we found that genes involved in processes linked to the regulation of Th2 cell differentiation and B-cell activation were up-regulated. It is currently proposed that *F. hepatica* produces a Th2 response or a down-regulation of Th1/Th17 that favours the longevity and survival of the parasite in the host animal [38,39]. The present results largely support this proposal (Figure 2). Some DTGs, for example *cd86* (cluster of differentiation 86 protein) and bcl6 (B-cell lymphoma 6 protein), are classic regulators of a Th2 response [76]. However, some data require careful interpretation and possibly further exploration. For example, we saw a significant up-regulation of *tgf*-*β1* and *tnf* genes in infected sheep. In mice, TGF-β1 promotes the development of Th17 cells [77,78], but at high concentrations, TGF-β1 has been shown to promote Fox3p + T-regulatory cells [76], which are anti-inflammatory [79] and a hallmark of many helminth infections [80]. Indeed, the suppression of inflammation is a common theme for many of the immune-related genes that we identified. For example, one of the few interleukins to be shown to be up-regulated in the current study (IL18BP) is a natural inhibitor of IFNγ and Th1 responses in humans [81] and suppresses inflammation associated with the Th17 response [82]. To our knowledge, this interleukin has not been studied in the interaction between liver flukes and their hosts. IL18BP production is stimulated in response to liver injury, and its increased concentration in serum is linked to the severity of liver damage [83]. Therefore, IL18BP should be explored as a biomarker for liver damage, investigating whether it correlates with the severity of fascioliasis. IL27RA was also up-regulated in infected sheep in the current study and is involved in signal transduction in response to IL27, which promotes Th1 differentiation and attenuates immune/inflammatory responses, suppressing Th17 differentiation and IL-17 production [84]. In contrast, TNFα regulates the release of pro-inflammatory cytokines, including IL1 [85] and was also up-regulated. However, intriguingly, one of the few interleukin-related genes that we found transcribed in the present study is *il1r2*; the protein encoded by this gene (IL1R2) acts a decoy receptor for IL1R1 and binds IL-1; in so doing, it prevents the release of a number of pro-inflammatory cytokines [86]. Thus, overall, the immune response in sheep against *Fasciola* does appear consistent with the ‘Th2 promotion and Th1/Th17 suppression’ model; however, the mechanisms underpinning this response are complex, potentially contradictory and would benefit from more detailed explorations at the specific cell-type level.

As observed in our histological study and those published in the literature [56,57,18], eosinophils are a major contributor to the cellular immune response against *Fasciola* in sheep. In the current study, we recorded an up-regulation of two galectin-9 isoforms (ENSOART00000001365 and ENSOART00000017447). One of them corresponds to LGALS9B in the sheep genome, which has been annotated previously as galectin-14 [87], is believed to be an eosinophil-specific galectin in sheep and cattle [87,88], and has been linked to host responses against *F. hepatica* infection [89]. Galectins bind glycans on the surface of potentially pathogenic microorganisms, and function as recognition and effector factors in innate immunity [90]. We found significant changes in transcription associated with the metabolism and biosynthesis of glycans in *Fasciola-*infected sheep. These changes might be the consequence of a rebalancing of the immune cell-types to achieve a Th2-biased response, with Th1/Th17 and Th2-cells having quite distinct surface glycan profiles [91]. The immune response against parasitic infection is not without implications for the host, particularly in relation to “collateral damage” caused by the induction of oxidative stress via the neutrophil/monocyte respiratory burst. Helminths have developed a number of strategies to counteract the host defences, including through the production of a cocktail of peroxidases and other anti-oxidant enzymes [92-94]. Host cells also need to deal with reactive oxygen species (ROS) released through respiratory bursts during the host immune response. The up-regulation in infected livers of numerous genes (e.g., *pxdn, il18bp, gpx3, prdx2, hp, col1a1, ect2, hbb* and *ada*) specifically involved in neutralizing ROS provides significant insight into the specific mechanisms through which sheep respond to the invading parasite and also the damage that the immune response can cause to host tissues as a consequence.

Many of the genes associated with *Fasciola*-infection straddle the boundary between stimulating an immune response and repairing tissue damage. For example, TLR4 and another, uncharacterized, TLR-related gene (ENSOART00000020222) were found to be up-regulated in infected tissue. Although, TLR4 is an important component of the innate immune system, it has also been connected with liver injury and hepatic fibrogenesis and other pathologies [95-98]. Additional examples are the up-regulation in infected animals of genes encoding the TGF-β1 and its Induced Transcript 1 (TGF-β1I1) as well as the chemokines CCL3 and CCL4. In addition to its roles in regulating T-cell differentiation (see above), TGF-β1 is also relevant in fibrosis [99-102]. CCL3 and CCL4 are expressed by a number of cell types, including activated B cells, monocytes, mast cells, fibroblasts and epithelial cells [103]. CCL3 has also been linked to both liver [104] and lung [105] fibrosis. The up-regulation of genes encoding phagocyte-derived S100s, specifically *s100a1*, *s100a12, s100a8* and *s100a9*, are also intriguing, because S100 proteins are endogenous activators of innate immune responses that mediate inflammatory responses and recruit inflammatory cells to sites of tissue damage [106,107]. They have also shown to be up-regulated in *Schistosoma japonicum*-induced hepatic granuloma [108] and are specifically linked to wound healing in a variety of tissues, including epidermis [109] and liver [110]. Notably, some of the S100 proteins up-regulated in infected livers have been highlighted as playing a key role in wool production in sheep [40]. Studies of sheep fascioliasis [111] have noted a 20-40% reduction in wool production, even in animals showing no overt signs of disease. Although the present data do not provide information on the transcription/expression of S100s outside of the liver, it is tempting to hypothesize that these S100 proteins are shunted from other tissues (e.g., the epidermis) to the liver in instances of trauma or infection, thus (alongside anaemia and other parasitic effects) effectively sacrificing wool production for liver repair.

The regeneration and repair of the tissue damaged by migrating juvenile *F. hepatica* are key components of the pathogenesis of fascioliasis. Notably, consistent with the high capacity of the liver to regenerate damaged tissue [112], we found a significant up-regulation in infected livers of genes associated with the cell cycle and mitosis (see Additional files 3 and 4). We also noted an up-regulation, relative to the uninfected controls, of *tnf*α, which contributes to the restoration of functional liver mass by driving hepatocyte proliferation and liver regeneration [113].

In addition to regenerating new tissue, the repair of existing liver tissue is critical to host survival. Central to the repair process is fibrosis, collagen deposition and the reformation of the extracellular matrix (ECM). Even at this relatively early stage of fascioliasis, we recorded a significant up-regulation of genes linked to the Jak-STAT pathway, which is known to play a major role in hepatic fibrosis [114]. In addition, we identified an up-regulation of a number of genes involved in these processes in vertebrates (Table 2 and Figure 3). Key examples of up-regulated genes linked to fibrosis were homologs of two *tgf-β*-related genes, insulin-like growth factor-binding protein-5 (IGFBP-5), calponin, transgelins (*tagln* and *tagln3*) and osteopontin (*ost*/*spp1*)*.* The role of TGF-β in fibrosis is well-documented [115] and mediated by IGFBP-5 [101]. Calponin and transgelins are expressed by hepatic stellate cells (HSCs) during liver fibrosis [116], with HSCs known to switch from vitamin A storage to a myofibroblast-like function in response to liver damage [117]. Calponin is induced in HSCs by TGF-β1 during liver fibrosis [118]. Osteopontin has been described as a regulator of T-helper-cell lineage development [119], but also drives fibrosis by promoting HSC activation and ECM deposition [120]. It has been suggested that blocking osteopontin could limit liver fibrosis and represents a potential target for ameliorating the pathogenicity of biliary trematodiases [121].

Key phases of fibrosis are collagen deposition and repair/remodelling of the ECM. In the present study, we found four collagen isoforms (*col1a1*, *col1a2*, *col16a1* and *col6a3*) to be up-regulated in infected sheep livers. We also observed an up-regulation of a number of key metalloproteases associated with *Fasciola* infection, including three disintegrins, four metalloproteinases (two isoforms each of *adam8* and *adam19*) and the genes encoding matrix metalloproteinases MMP19 and TIMP1. Metalloproteinases are ECM-degrading enzymes, often expressed in the liver by HSCs in response to diverse hepatic toxins and/or damage [122,123]. Tissue and ECM remodelling is likely also regulated by serine protease inhibitors, with *serpinb8* and *serpini1* both up-regulated in infected sheep. Serpins are important in a variety of cell types and are up-regulated during injury repair [124,125]. In addition to their expression in most (healthy) tissues, they can be found in immune cells [126], highlighting again the dual-function of many of the DTGs identified in this study. Adenosine receptor A2b (*adora2b*), a key regulator of ECM formation and collagen deposition by fibroblasts [127], was found to be up-regulated. This gene plays a role in blocking liver injury [128], suggesting that it regulates fibrosis in sheep. Considering that fibrosis is often limited in cases of chronic fascioliasis in sheep compared with cattle [3], it would be interesting to confirm the role of *adora2b* during infection by studying its expression in cattle.

## Conclusions

The current study is, to our knowledge, the first detailed investigation of the transcriptomic responses in the liver tissue of any host to *F. hepatica* infection. This study defines the involvement of specific genes associated with the host’s metabolism, immune response and tissue repair/regeneration, and highlights the apparent overlapping function of many of the genes involved in these processes. Of major relevance is the characterization of genes involved in suppressing the Th1/Th17 response (e.g., genes encoding IL18BP and IL27RA), likely contributing to the Th2 biased response against *F. hepatica* infection, widely reported in the literature. Not surprisingly, genes associated with fibrosis and tissue repair, remodelling and regeneration feature prominently among up-regulated genes in the infected animals. The present data highlight a role for a number of genes, including *tnf-α*, *tgf-β*, *calponins, transgelins, osteopontin* and *adora2b*, associated with these particular functions. Considering the unique manifestation of fascioliasis in cattle compared with sheep, particularly in relation to the extent of the fibrotic response during the chronic stage of infection, detailed exploration of *Fasciola*-infection of cattle would be highly relevant and would likely provide insight into distinctiveness of disease pathogenesis in these animals. Interestingly, aside from *il27ra*, *il18bp* and *il1r2*, we found no evidence for the transcription of a wide-variety of interleukins in any (control or infected) sheep studied. Their absence from our data might suggest that our approach was not appropriate for their detection, and we thus make no inference regarding their involvement in fascioliasis in sheep at this stage. Although we studied here liver with clear signs of tissue damage linked to the migration of juvenile flukes, it may be that the transcription of many key interleukins and other molecules associated with *Fasciola* infection in the liver were too localized or ephemeral for detection using the methods employed here. Subsequent studies may benefit from employing laser micro-dissection and tissue capture [129], targeting tissue(s) in intimate contact with the parasite. Such studies would also allow joint investigations of transcription in the host cells as well as individual parasites in direct contact with each other; this likely represents a powerful approach for elucidating, in great detail, host-parasite interactions. Further studies directed at exploring changes to host transcriptional behaviour over the duration of liver invasion and the establishment of adult *Fasciola* in the bile duct, for example, by exploring transcription in peripheral blood mononuclear cells (PBMCs) and/or the lymphatic vessels draining the liver, would be highly informative.

## Additional files

Additional file 1: Table S1. Total number of reads per sample from infected and control replicates. Additional file 2: Table S2. Full list of 572 up-regulated genes in infected liver tissue. **Table S3.** Full list of 42 down-regulated genes in infected liver tissue. Additional file 3: Table S4. List of overrepresented GO terms related with “biological Processes” for up-regulated genes in infected liver tissue. **Table S5.** List of overrepresented GO terms related with “molecular function” for up-regulated genes in infected liver tissue. **Table S6.** List of overrepresented GO terms related with “Cellular component” for up-regulated genes in infected liver tissue. **Table S7.** List of overrepresented GO terms related with “biological processes” for down-regulated genes in infected liver tissue. **Table S8.** List of overrepresented GO terms related with “molecular function” for down-regulated genes in infected liver tissue. **Table S9.** List of overrepresented GO terms related with “cellular component” for down-regulated genes in infected liver tissue. Additional file 4: Table S10. List of enriched KEGG pathways for up-regulated genes in infected liver tissue. **Table S11.** List of enriched KEGG pathways for down-regulated genes in infected liver tissue.
