# Supplementary material for: Transcriptional analysis identifies key genes involved in metabolism, fibrosis/tissue repair and the immune response against Fasciola hepatica in sheep liver
Source: Parasit Vectors. 2015 Feb 25;8:124. doi: 10.1186/s13071-015-0715-7 (PMC4382932; doi:10.1186/s13071-015-0715-7)
Supplement: Additional file 1: Table S1. — Total number of reads per sample from infected and control replicates. [file 13071_2015_715_MOESM1_ESM.pdf]

Reads	Infected replicates ID INF-1	INF-2	INF-3	INF-4
Raw	11,413,396	27,579,489	12,250,158	11,353,964
After correction	8,416,710	21,863,048	7,903,548	8,756,200
After trimming	7,497,036	20,082,000	7,341,132	8,171,910
Mapped	5,146,533	13,689,041	5,137,052	5,539,721
% mapped	68.7	68.1	70	67.8
Mean coverage X	25.3	65.6	20.3	20.3

Reads	Control replicates ID CON-1	CON-2	CON-3	CON-4
Raw	18,947,055	9,648,569	20,896,769	11,315,973
After correction	13,197,272	6,753,998	15,681,382	9,218,044
After trimming	11,659,834	5,678,864	13,730,146	8,397,022
Mapped	8,308,832	2,281,982	9,773,966	5,504,993
% mapped	71.2	63.2	71.2	65.5
Mean coverage X	35.5	13	46.9	30.6

Additional file 1, Table S1. Total number of reads per sample for infected and control replicates: raw data, after k-mer correction, after trimming, number of reads mapped to the sheep genome Oar_v3.1 using RSEM and sequencing coverage for each sample in the infected (INF) and control group (CON).
